# A novel bacterial β-*N*-acetyl glucosaminidase from *Chitinolyticbacter meiyuanensis* possessing transglycosylation and reverse hydrolysis activities

**DOI:** 10.1186/s13068-020-01754-4

**Published:** 2020-06-29

**Authors:** Alei Zhang, Xiaofang Mo, Ning Zhou, Yingying Wang, Guoguang Wei, Jie Chen, Kequan Chen, Pingkai Ouyang

**Affiliations:** State Key Laboratory of Materials-Oriented Chemical Engineering, College of Biotechnology and Pharmaceutical Engineering, Nanjing Tech University, Nanjing, 211800 People’s Republic of China

**Keywords:** β-*N*-acetyl glucosaminidase, *N*-Acetyl glucosamine, *N*-Acetyl chitooligosaccharides, Exo-acting activity, Transglycosylation, Reverse hydrolysis

## Abstract

**Background:**

*N*-Acetyl glucosamine (GlcNAc) and *N*-Acetyl chitooligosaccharides (*N*-Acetyl COSs) exhibit many biological activities, and have been widely used in the pharmaceutical, agriculture, food, and chemical industries. Particularly, higher *N*-Acetyl COSs with degree of polymerization from 4 to 7 ((GlcNAc)_4_–(GlcNAc)_7_) show good antitumor and antimicrobial activity, as well as possessing strong stimulating activity toward natural killer cells. Thus, it is of great significance to discover a β-*N*-acetyl glucosaminidase (NAGase) that can not only produce GlcNAc, but also synthesize *N*-Acetyl COSs.

**Results:**

The gene encoding the novel β-*N*-acetyl glucosaminidase, designated *Cm*NAGase, was cloned from *Chitinolyticbacter meiyuanensis* SYBC-H1. The deduced amino acid sequence of *Cm*NAGase contains a glycoside hydrolase family 20 catalytic module that shows low identity (12–35%) with the corresponding domain of most well-characterized NAGases. The *Cm*NAGase gene was highly expressed with an active form in *Escherichia coli* BL21 (DE3) cells. The specific activity of purified *Cm*NAGase toward *p*-nitrophenyl-*N*-acetyl glucosaminide (*p*NP-GlcNAc) was 4878.6 U/mg of protein. *Cm*NAGase had a molecular mass of 92 kDa, and its optimum activity was at pH 5.4 and 40 °C. The *V*_max_, *K*_m_, *K*_cat_, and *K*_cat_/*K*_m_ of *Cm*NAGase for *p*NP-GlcNAc were 16,666.67 μmol min^−1^ mg^−1^, 0.50 μmol mL^−1^, 25,555.56 s^−1^, and 51,111.12 mL μmol^−1^ s^−1^, respectively. Analysis of the hydrolysis products of *N*-Acetyl COSs and colloidal chitin revealed that *Cm*NAGase is a typical exo-acting NAGase. Particularly, *Cm*NAGase can synthesize higher *N*-Acetyl COSs ((GlcNAc)_3_–(GlcNAc)_7_) from (GlcNAc)_2_–(GlcNAc)_6_, respectively, showed that it possesses transglycosylation activity. In addition, *Cm*NAGase also has reverse hydrolysis activity toward GlcNAc, synthesizing various linked GlcNAc dimers.

**Conclusions:**

The observations recorded in this study that *Cm*NAGase is a novel NAGase with exo-acting, transglycosylation, and reverse hydrolysis activities, suggest a possible application in the production of GlcNAc or higher *N*-Acetyl COSs.

## Background

Chitin, a linear polysaccharide of 1, 4-β-linked *N*-Acetyl glucosamine (GlcNAc), is the second most abundant renewable source in nature behind cellulose and mainly exists in crustacean shells, fungal cell walls, and insect exoskeletons [1]. Comprehensive utilization of these chitin biomasses may have economic and ecological benefits [2]. However, there is no satisfactory method to utilize them to date. A vast majority of the chitin biomasses are directly disposed of or landfilled without utilization, which leads to serious pollution and wasted resources [3]. Therefore, it would be beneficial to utilize the numerous chitin resources to produce value-added chemicals and materials.

Chitin can be used to produce biomaterials such as films, adhesives, preservative coatings, and antibacterial/anticancer materials [4–6]. Besides the direct utilization of chitin mentioned above, chitin can be used as a substrate for producing nitrogen-containing chemicals like GlcNAc, *N*-Acetyl chitooligosaccharides (*N*-Acetyl COSs), ethanolamine, N-containing furan derivatives and so on [2]. Among these chemicals, GlcNAc and *N*-Acetyl COSs are considered promising platform molecules, and have been widely used in the pharmaceutical, agriculture, food, and chemical industries [7, 8]. Especially, higher *N*-Acetyl COSs with the degree of polymerization from 4 to 7 ((GlcNAc)_4_–(GlcNAc)_7_) exhibit many biological activities. For example, (GlcNAc)_4_ was found to have strong stimulating activity toward natural killer cells [9]. (GlcNAc)_5_ is an important building block for NOD factor synthesis [10]. (GlcNAc)_6_ and (GlcNAc)_7_ show antitumor activity against mice sarcoma 180 [11] and antimicrobial activity against fungal pathogens [12].

Chitin is traditionally chemically degraded to GlcNAc and *N*-Acetyl COSs using acid, which leads to toxicity and risks associated with serious pollution during the production process [13]. With increased environmental awareness, increasing attention has been paid to developing enzymatic hydrolysis of chitin using chitinolytic enzymes as catalysts because they are environmentally friendly and result in products with high bioactivity compared to classical chemical routes [14].

Chitinolytic enzymes, the essential enzymes involved in catabolism of chitin, primarily include chitinase [mainly belonging to glycoside hydrolase (GH) families 18 and 19, hydrolyzes chitin to *N*-Acetyl COSs] [15], β-*N*-acetyl glucosaminidase (GH families 3, 20, 73, 84, and 85, hydrolyzes *N*-Acetyl COSs to GlcNAc) [16, 17], and lytic polysaccharide monooxygenase [Auxiliary Activity (AA) families 10 and 11, cleavage of chitin chains with oxidation to enhance the hydrolysis of chitin] [18]. Of these, β-*N*-acetyl glucosaminidase (NAGase), an indispensable member of the chitinolytic system, has a significant physiological role depending on its origin. With the cooperative action of NAGase and chitinase, chitin can be hydrolyzed into GlcNAc. Moreover, some GH20 NAGases can also be used to synthesize high-value GlcNAc-containing products and *N*-Acetyl COSs by transglycosylation and reverse hydrolytic reactions [19–24]. These excellent features make GH20 NAGases receive increased attention. GH20 NAGases can be found in a wide variety of organisms including bacteria, fungal, insects, plants, and animals [24–27]. However, studies about the GH20 NAGases with transglycosylation and reverse hydrolysis activities are mainly derived from fungal sources [28–34]. There are few reports about the bacterial NAGases possessing transglycosylation and reverse hydrolysis activities [19, 21, 35, 36].

In our previous study, a chitinolytic bacterium *Chitinolyticbacter meiyuanensis* SYBC-H1 with a good ability to degrade chitin was isolated from soil [37]. In this study, a gene encoding NAGase was cloned from the SYBC-H1 strain, based on the results of peptide mass fingerprinting and complete genome sequencing, and heterologously expressed in *Escherichia coli* BL21(DE3). The phylogenetic relationships and catalytic characteristics of the purified recombinant NAGase were described. Furthermore, its transglycosylation and reverse hydrolysis activities were also investigated.

## Results and discussion

### Purification of wild-type NAGase from *C. meiyuanensis* SYBC-H1

**Fig. 1:**
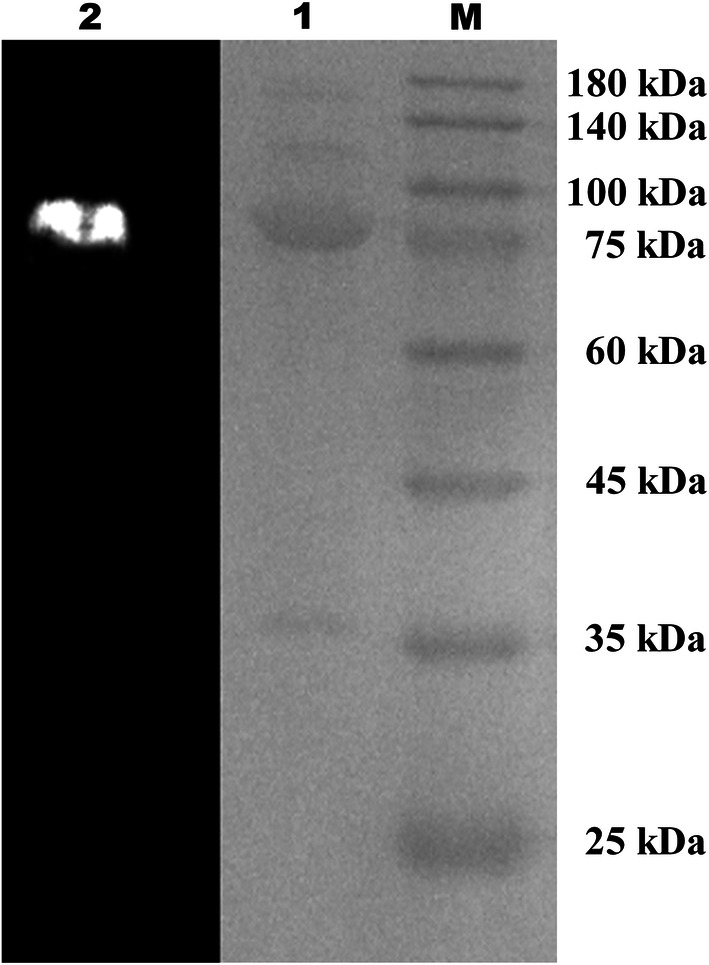
Zymogram analysis of the purified *Cm*NAGase. The purified enzyme was analyzed by SDS-PAGE using a 3% stacking gel and a 10% separating gel with addition of SDS. Lane M, protein molecular mass makers; lane 1, purified *Cm*NAGase (20 μg) visualized by Coomassie brilliant blue staining; lane 2, purified *Cm*NAGase staining with 4-MU-GlcNAc and visualized at 340 nm

Purification of the NAGase from *C. meiyuanensis* SYBC-H1 was performed. Following ammonium sulfate precipitation, anion exchange chromatography, and SDS-PAGE, a protein band with NAGase activity was obtained, as shown by zymogram analysis. The band with NAGase activity (named *Cm*NAGase) had a molecular mass of between 75 and 100 kDa (Fig. 1).

The protein strip in the stained gel was excised for peptide mass fingerprinting (PMF) analysis using matrix-assisted laser desorption ionization-time-of-flight (MALDI-TOF MS/MS), and the results of PMF were interpreted by referencing the Mascot database [38]. Proteins receiving the highest molecular weight search scores (MOWSE) were selected as the peptide fragments of purified protein. Peptide fragments of purified protein were mainly detected with the amino acid sequences of YDGDTFLARLTLTNH, AMNVRYERLVKAGK, and WNQFANRLGQRELARLDGFLGGYGYRVPV, which showed 100% identity to the peptides from an annotated NAGase in the complete genome of *C. meiyuanensis* SYBC-H1.

### Cloning of the *Cm*NAGase gene and sequence analysis

The *Cm*NAGase gene was cloned. As expected, a PCR product of 2.5 kb was obtained. Analysis of the PCR product showed that the *Cm*NAGase gene was 2508 bp, encoding for a protein of 836 amino acids. The calculated molecular mass of *Cm*NAGase was 91.6 kDa, and the isoelectric point (pI) was predicted to be 5.48. The sequence analysis suggested no putative signal peptide in the sequence of *Cm*NAGase, which suggested that *Cm*NAGase should be a non-secretory protein. The prediction using Gneg-mPLoc 2.0 also showed that the location of *Cm*NAGase was in the periplasm. However, *Cm*NAGase could be purified from the fermentation broth of the strain SYBC-H1. Furthermore, most reported NAGases are secretory proteins to date [21–23]. These results show that the prediction of *Cm*NAGase location may be wrong.

**Fig. 2:**
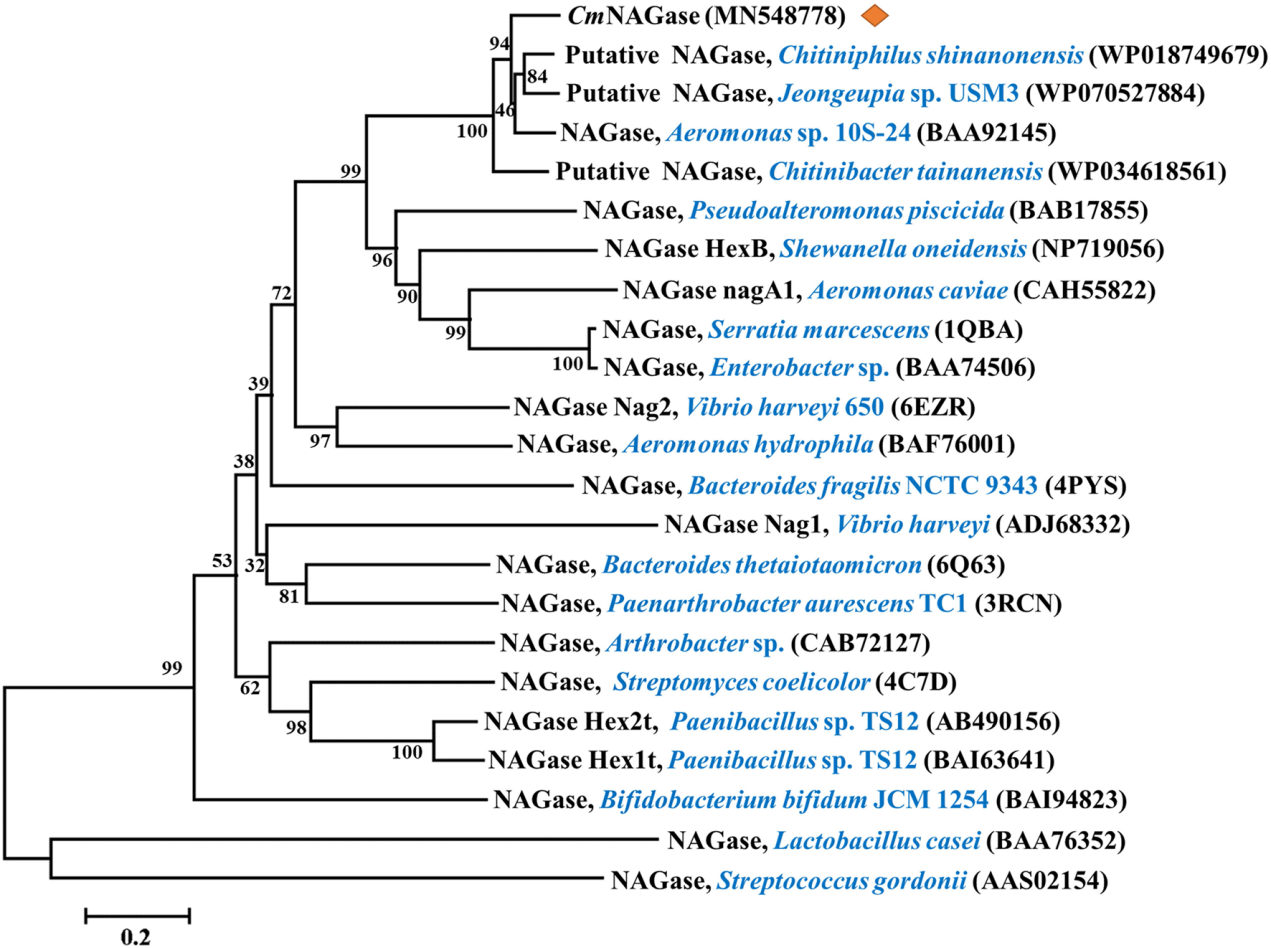
Phylogenetic relationships between *Cm*NAGase and other bacterial NAGases from the glycoside hydrolase (GH) family 20. The phylogenetic tree was constructed by the neighbor-joining algorithm based on the amino acid sequence alignment in MEGA 7.0. The amino acid sequence of *Cm*NAGase was aligned with those of the following proteins: GH20 NAGases from *Chitiniphilus shinanonensis* (WP_018749679), *Jeongeupia* sp. USM3 (WP_070527884), *Aeromonas* sp. 10S-24 (BAA92145), *Chitinibacter tainanensis* (WP_034618561), *Pseudoalteromonas piscicida* (BAB17855), *Shewanella oneidensis* (HexB, NP719056), *Aeromonas caviae* (nagA1, CAH55822)*, Serratia marcescens* (PDB 1QBA)*, Enterobacter* sp. (BAA74506), *Vibrio harveyi* 650 (Nag2, PDB 6EZR), *Aeromonas hydrophila* (BAF76001)*, Bacteroides fragilis* NCTC 9343 (PDB 4PYS), *V. harveyi* (Nag1, ADJ68332), *Bacteroides thetaiotaomicron* (PDB 6Q63), *Paenarthrobacter aurescens* TC1 (PDB 3RCN), *Arthrobacter* sp. (CAB72127), *Streptomyces coelicolor* (4C7D), *Paenibacillus* sp. TS12 (Hex2t, AB490156), *Paenibacillus* sp. TS12 (Hex1t, BAI63641), *Bifidobacterium bifidum* JCM 1254 (BAI94823), *Lactobacillus casei* (BAA76352), and *Streptococcus gordonii* (AAS02154)

According to the Carbohydrate-Active enZYmes (CAZy) database (http://www.cazy.org/), NAGases can be classified as part of the glycoside hydrolase (GH) families 3, 20, 73, 84, and 85 based on amino acid sequence homology. BLASTP analysis showed that *Cm*NAGase belonged to GH family 20 (GH20) and shared the highest identity (81.68%) with the GH20 NAGase ChiI from *Chitiniphilus shinanonensis* (WP_018749679) [39], followed by GH20 NAGase (81.44%) from *Chitiniphilus* sp. HX-2-15 (WP_136772659). However, these coding genes have not been expressed and studied. Among characterized GH20 NAGases, *Cm*NAGase showed the highest identity (77.58%) with GH20 NAGase from *Aeromonas* sp. 10S-24 (Accession no. BAA92145) [40], following by the GH20 NAGase (34.02%) from *Serratia marcescens* (PDB 1QBA) [41], GH20 NAGase (30.44%) from *Aeromonas caviae* CB101 (Accession no. CAH55822) [42], GH20 NAGase Nag2 (30.04%) from *Vibrio harveyi* (PDB 6EZR) [43], GH20 NAGase (29.01%) from *Enterobacter* sp. G-1 (Accession no. BAA74506) [44], and GH20 NAGase (24.64%) from *Aeromonas hydrophila* SUWA-9 (Accession no. BAF76001) [45], GH20 NAGase Nag1 (12.26%) from *V. harveyi* (Accession no. ADJ68332) [43], and GH20 NAGase (12.24%) from *Arthrobacter* sp. TAD20 (Accession no. CAB72127) [46]. The putative GH20 NAGases with the highest similarity of *Cm*NAGase and verified GH20 NAGases were performed to construct the phylogenic tree, which also showed that *Cm*NAGase exhibited a low sequence identity (12–35%) with most of functionally characterized bacterial GH20 NAGases (Fig. 2).

GH20 NAGases employ the retaining mechanism for catalysis. The enzymes carry out substrate-assisted catalysis, in which a Glu (E509 in *Cm*NAGase) acts as the general acid/base residue for protonation, while Asp (D508 in *Cm*NAGase) acts to orient the C2-acetamido group into position for correct nucleophilic attack by a water molecule, and subsequently provides the negatively charged carboxylate groups to stabilize the positively charged oxazolinium ion intermediate [26]. Multiple alignments of the catalytic domain in *Cm*NAGase with other GH20 NAGases from different sources indicated the substrate-binding residues (R315, H422, V463, Q464, W558, W594, Y621, D623, L624, Y635, W637, W693, and E695) and catalytic residues (D508 and E509) in *Cm*NAGase, which are highly conserved among GH20 members (Additional file 1: Fig. S1). Moreover, sequence ^504^H/N-X-A/C/G/M-D-E-A/I/L/V^510^ in *Cm*NAGase is the highly conserved amino acid sequence in the catalytic domain of GH20 NAGases from bacteria, fungi, and archaea [47]. The analysis of secondary structure showed that *Cm*NAGase possesses 21 α-helices and 31 β-sheets with the typical TIM-barrel(β/α)_8_ fold in the GH20 catalytic domain (Additional file 1: Fig. S1), which is consistent with various GH20 NAGases from different sources [22].

Domain structure prediction revealed that *Cm*NAGase contains four domains: CHB_HEX domain of residues 7–155 (putative carbohydrate binding domain); Glyco_hydro_20b domain of residues 174–300 (N-terminal domain of the beta-hexosaminidases); Glyco_hydro_20 domain of residues 304–719 (catalytic domain); and CHB_HEX C_1 domain of residues 753–828 (unknown function) (Additional file 1: Fig. S2a). As shown in Additional file 1: Fig. S2b, the 3D structure of *Cm*NAGase was predicted based on the structure model of 1QBA (34.02%) [41]. The active sites (R315, H422, V463, Q464, D508, E509, W558, W594, Y621, D623, L624, Y635, W637, W693, and E695) were also shown in the 3D structure of *Cm*NAGase (Additional file 1: Fig. S2c), and form an active pocket [47].

### Expression of *Cm*NAGase gene and purification of recombinant *Cm*NAGase

The full-length of *Cm*NAGase gene was successfully expressed in *E. coli* BL 21 (DE3) at a high expression level (~ 50% of total protein). The recombinant *Cm*NAGase with a C-terminal His6-tag was purified by Ni-NTA affinity chromatography with a yield of 80.5%. The reason for the high purification yield may be that the C-terminal His6-tag in the recombinant *Cm*NAGase was well exposed, which led to the higher affinity with Ni-NTA resin. The specific activity of recombinant *Cm*NAGase increased 1.5-fold from 3156.5 U/mg to 4878.6 U/mg after purification (Additional file 1: Table S2). The SDS-PAGE analysis showed that purified recombinant *Cm*NAGase possesses a high purity with an approximate molecular weight of 92 kDa, which agrees with 92,571 kDa calculated from the amino acid sequence containing the His6-tag (Additional file 1: Fig. S3).

### Effects of pH and temperature on activity and stability of recombinant *Cm*NAGase

**Fig. 3:**
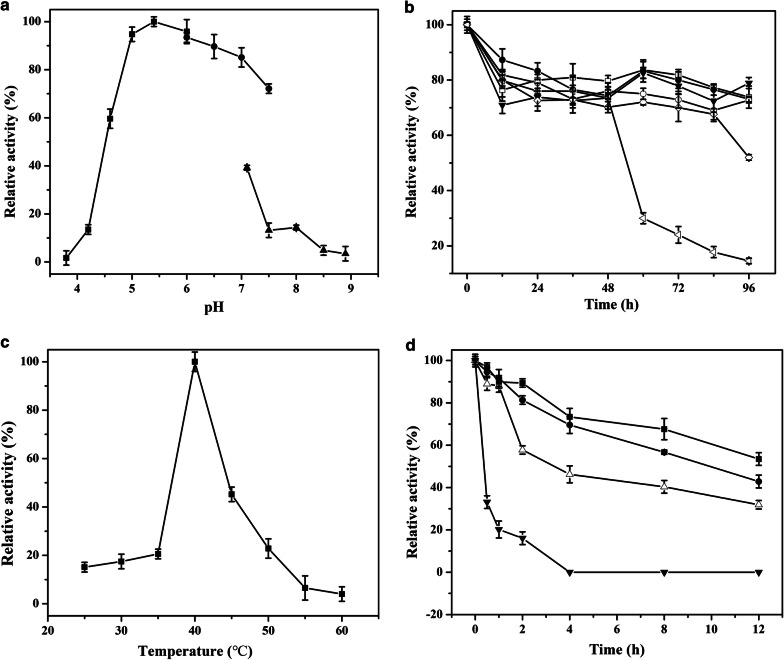
Effect of temperature and pH on *Cm*NAGase. **a** The optimal pH of *Cm*NAGase. The optimal pH was determined in 50 mM solutions of various buffers within the pH range of 3.5–10.0 [filled square, citrate buffer (pH 3.0–6.0); filled circle, phosphate buffer (pH 6.0–7.5); filled inverted triangle, Tris–HCl buffer (pH 7.0–9.0)]. **b** The stability of *Cm*NAGase at various pH values. To determine pH stability, the enzyme was incubated at 35 °C for 96 h with various pH buffers, and the residual activities were measured [filled inverted triangle, 4.7 (citrate buffer); open square, 5.4 (citrate buffer); open diamond, 6.0 (citrate buffer); open circle, 7.0 (phosphate buffer); filled circle, 8.0 (Tris–HCl buffer); open square, 9.0 (Tris–HCl buffer)]. **c** The optimal temperature of *Cm*NAGase. The temperature optimum was determined at different temperatures (25–60 °C) in 50 mM sodium citrate (pH 5.4) of the recombinant *Cm*NAGase. **d** The stability of *Cm*NAGase at different temperatures. To determine the thermostability, the enzyme was treated in 50 mM sodium citrate (pH 5.4) for 12 h at different temperatures; the residual activity was measured at pH 5.4 and 40 °C (filled square, 30 °C; filled circle, 35 °C; open triangle 40 °C; filled inverted triangle, 45 °C)

The pH and temperature profile of *Cm*NAGase activity are shown in Fig. 3. Typically, the optimal pH of reported GH20 NAGases is in the range of pH 5.0 to pH 8.0. *Cm*NAGase exhibited a high level of activity at pH 4.0–7.0 with the optimal pH of 5.4 (Fig. 3a), which is different from that of NAGases from *Aeromonas* sp. 10S-24 (7.0) [40], *Paenibacillus* sp. TS12 (6.0) [48], *Vibrio harveyi* 650 (7.5) [43], *Enterobacter* sp. G-1 (6.0) [44], *Salmonella enterica* (4.0) [49], *Paraglaciecola hydrolytica* S66 (6.0) [21], and *Cellulomonas fimi* (7.3–8.7) [50]. In addition, *Cm*NAGase presented good activity after being stored at pH 4.0–8.5 for more than 84 h (Fig. 3b), which suggested that the *Cm*NAGase possesses a good pH stability compared with other reported NAGases [45, 51, 52].

As shown in Fig. 3c, the effect of temperature on enzymatic activity showed that the optimal temperature of *Cm*NAGase was 40 °C, which is different from that of NAGases from *Shinella* sp. (50 °C) [27], *Trichoderma reesei* (60 °C) [51], *P. hydrolytica* S66 (50 °C) [21], *Enterobacter* sp. G-1 (45 °C) [44], and *S. marcescens* (52 °C) [53]. The *Cm*NAGase was unstable at temperatures ˃ 40 °C (Fig. 3d). Half-lives of *Cm*NAGase toward 30 °C, 35 °C, 40 °C, and 45 °C were 13.0, 9.5, 6.3, and 0.6 h, respectively (Additional file 1: Table S3), which were similar with that of GH20 NAGases from *Lactobacillus casei* [54], *Sphingobacterium* sp. [55], and *Aeromonas* sp. 10S-24 [40]. These results suggested that *Cm*NAGase is a mesophilic and acidic enzyme.

### Effect of metal ions on activity of recombinant *Cm*NAGase

The effects of metal ions on *Cm*NAGase were investigated. All counter-ions of the used metal ions were Cl^−^. As shown in Table 1, EDTA did not inhibit the enzymatic activity, which indicates that *Cm*NAGase is not metal-dependent. *Cm*NAGase activity is completely inhibited by Zn^2+^, Cu^2+^, and Al^3+^, severely inhibited by Ba^2+^, Fe^3+^, and Cr^3+^. To date, many studies have shown that Zn^2+^, Cu^2+^, Fe^3+^, and Al^3+^ inhibit the activity of NAGases. For example, the GH20 NAGase from *A. caviae* is strongly inhibited by Cu^2+^ and Zn^2+^ [42]; the GH20 NAGase from *Paenibacillus* sp. is strongly inhibited by Zn^2+^ [48], and the GH20 NAGase from *T. reesei* is partially inhibited by Fe^3+^ [51]. Mn^2+^ enhanced the activity of *Cm*NAGase, which is different from the GH20 NAGase from *A. caviae* (strongly inhibited by Mn^2+^) [42]. However, the specific activated mechanism of Mn^2+^ is unclear and the in-depth study is needed in the future.

**Table 1:** Effect of different metal ions on *Cm*NAGase

Metal ions	Chemicals	Relative activity (%)
No addition	–	100
EDTA	EDTA	101.4 ± 3.2
Na^+^	NaCl	96.8 ± 6.7
K^+^	KCl	101.9 ± 7.8
Fe^2+^	FeCl_2_	97.6 ± 4.2
Ca^2+^	CaCl_2_	93.9 ± 6.6
Cu^2+^	CuCl_2_	0
Ni^2+^	NiCl_2_	106.3 ± 4.9
Zn^2+^	ZnCl_2_	0
Mg^2+^	MgCl_2_	83.3 ± 6.3
Mn^2+^	MnCl_2_	116.3 ± 3.5
Co^2+^	CoCl_2_	103.1 ± 3.8
Ba^2+^	BaCl_2_	34.7 ± 4.6
Al^3+^	AlCl_3_	0
Fe^3+^	FeCl_3_	21.3 ± 1.1
Cr^3+^	CrCl_3_	4.3 ± 2.5

Samples were preincubated with various mental ions (10 mM) at pH 7.0 (Tris–HCl buffer) and 4 °C for 30 min. The remaining activity was measured with *p*NP-GlcNAc at pH 7.0 (Tris–HCl buffer) and 40 °C for 10 min. Activity in the absence of any additives was taken as 100%

### The substrate specificity of recombinant *Cm*NAGase

The specific activities of the recombinant *Cm*NAGase against various substrates were investigated using standard assay conditions. As shown in Table 2, *Cm*NAGase can hydrolyze *p*NP-GlcNAc, 4-MU-GlcNAc, and (GlcNAc)_2_–(GlcNAc)_6_. No activity was observed when *p*NP-glucose, *p*NP-acetyl galactosaminide, and cellobiose were used as the substrates. These results showed that *Cm*NAGase represents the typical NAGase activity with strict substrate specificity. The specific activity of *Cm*NAGase toward *p*NP-GlcNAc can reach 4878.6 U/mg, which is higher than most reported NAGases (< 2000 U/mg) [17, 21, 27, 48, 56].

**Table 2:** Substrate specificity of *Cm*NAGase

Substrates^a^	Specific activity (U/mg of protein)
Colloidal chitin	0.02 ± 0.001
*p*NP-GlcNAc	4878.6 ± 200.8
*p*NP-Glucoside	–
*p*NP-Acetylgalactosaminide	–
4-MU-GlcNAc	4206.1 ± 283.8
Cellobiose	–
(GlcNAc)_2_	5305.4 ± 125.6
(GlcNAc)_3_	3132.8 ± 280.5
(GlcNAc)_4_	1409.4 ± 67.3
(GlcNAc)_5_	1116.2 ± 45.1
(GlcNAc)_6_	473.7 ± 36.8

–: Activity was not detected

^a^Reaction mixture (1 mL) containing enzyme (3 μg) and substrates (10 g/L) was incubated at pH 5.4 and 40 °C for 10 min

Most of the GH20 NAGases have the highest catalytic efficiency for (GlcNAc)_2_ among natural substrates, and do not hydrolyze *N*-acetyl COSs and chitin polymer [22, 56]. However, *Cm*NAGase showed good activities toward (GlcNAc)_2_–(GlcNAc)_6_ with the highest activity for (GlcNAc)_2_, followed by (GlcNAc)_3_, (GlcNAc)_4_, (GlcNAc)_5_, and (GlcNAc)_6_. These results show that specific activity of *Cm*NAGase toward *N*-Acetyl COSs decreased when the degree of polymerization increased, which is similar with other reports [57]. The catalytic efficiency of GH20 *Le*Hex20A from *Lentinula edodes* for (GlcNAc)_6_ was greater than for (GlcNAc)_2_ [58]. The GH20 NAGase *Vh*Nag2 from *V. harveyi* showed the highest activity against (GlcNAc)_4_, while the lowest activity was observed with (GlcNAc)_2_ [59]. These NAGases are different from *Cm*NAGase. Furthermore, *Cm*NAGase showed some activity (0.02 U/mg) toward colloidal chitin. The result is similar with the GH20 NAGases from *V. harveyi* [59] and the fungal NAGases from *Myceliopthora thermophila* [60], *L. edodes* [58], and *M. anisopliae* [61], which were reported to degrade chitin to some extent without the cooperation of chitinase.

In addition, the kinetic parameters for *Cm*NAGase were also measured with *p*NP-GlcNAc as the substrate. The results showed that the *V*_max_, *K*_m_, *K*_cat_, and *K*_cat_/*K*_m_ for *Cm*NAGase were 16,666.67 μmol min^−1^ mg^−1^, 0.5 μmol mL^−1^, 25,555.56 s^−1^, and 51,111.12 mL μmol^−1^ s^−1^, respectively.

### Hydrolysis reaction of recombinant *Cm*NAGase toward colloidal chitin

**Fig. 4:**
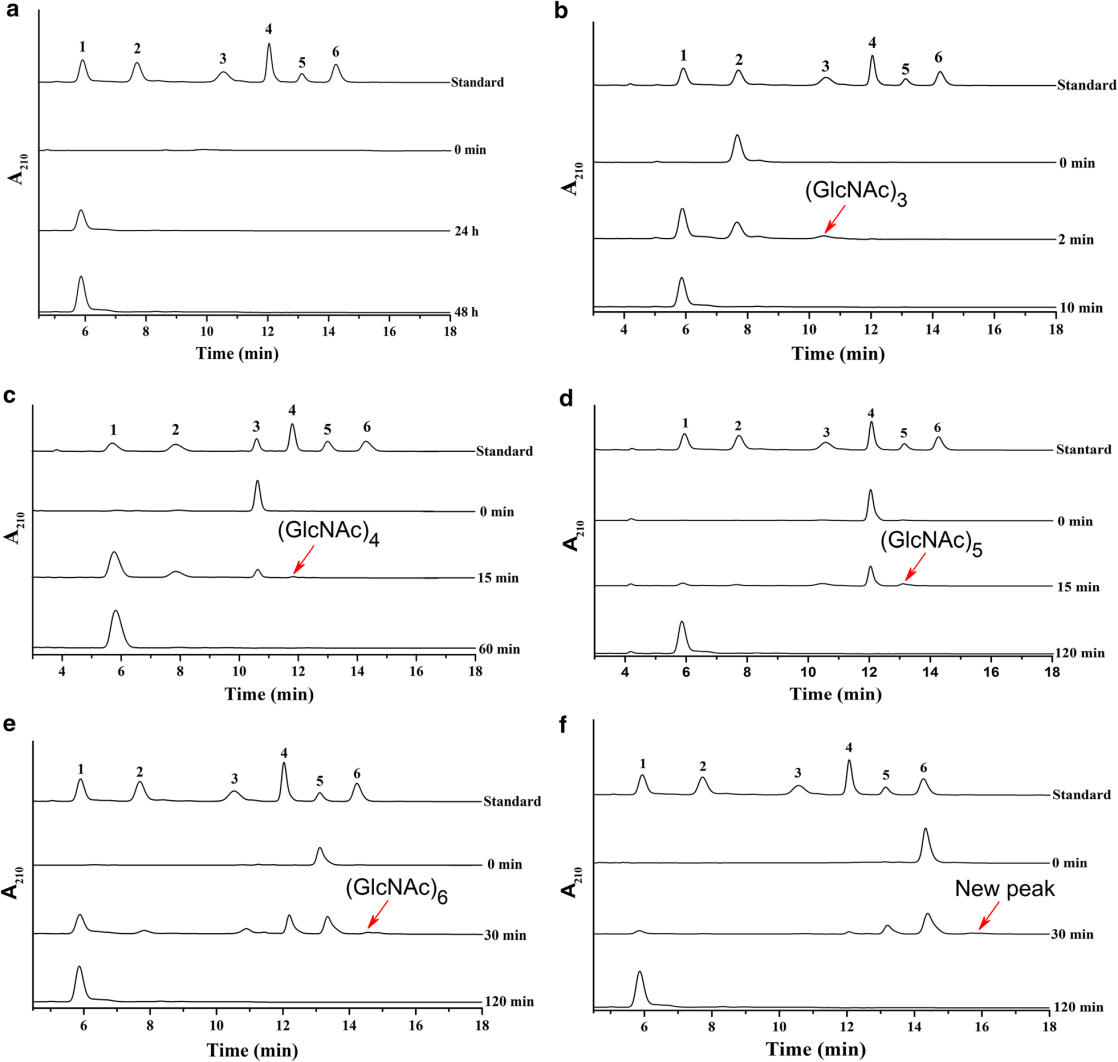
HPLC analysis of the hydrolysate of colloidal chitin and *N*-Acetyl COSs by *Cm*NAGase. **a** Colloidal chitin and **b**–**e** (GlcNAc)_2_–(GlcNAc)_6_ were incubated with *Cm*NAGase (0.3 μg) in 100 μL volume at pH 5.4 and 35 °C for different time intervals. 1–6: Standards of GlcNAc, (GlcNAc)_2_, (GlcNAc)_3_, (GlcNAc)_4_, (GlcNAc)_5_, and (GlcNAc)_6_

As shown in Fig. 4a, hydrolysis of colloidal chitin resulted in GlcNAc as the only product, and its concentration increased with the increase of hydrolysis time. Konno et al. [58] reported that the NAGase *Le*Hex20A from *L. edodes* hydrolyzes colloidal chitin to various *N*-acetyl COSs at the start of the reaction, and these *N*-Acetyl COSs convert to GlcNAc after 3 h, which is different from *Cm*NAGase.

### Hydrolysis and transglycosylation reactions of recombinant *Cm*NAGase toward *N*-Acetyl COSs

To evaluate the hydrolysis and transglycosylation activities of *Cm*NAGase, (GlcNAc)_2_–(GlcNAc)_6_ were used as substrates. The overall rates of hydrolysis were in the order: (GlcNAc)_2_ > (GlcNAc)_3_ > (GlcNAc)_4_ > (GlcNAc)_5_ > (GlcNAc)_6_, which is consistent with the results of substrate specificities above. (GlcNAc)_2_ was degraded by *Cm*NAGase to GlcNAc (Fig. 4b). GlcNAc and (GlcNAc)_2_ are produced from (GlcNAc)_3_ (Fig. 4c). When using (GlcNAc)_4_ as a substrate, GlcNAc, (GlcNAc)_2_, and (GlcNAc)_3_ were produced (Fig. 4d). GlcNAc, (GlcNAc)_2_, (GlcNAc)_3_, and (GlcNAc)_4_ were obtained from (GlcNAc)_5_ (Fig. 4e). When using (GlcNAc)_6_ as the substrate, GlcNAc, (GlcNAc)_2_, (GlcNAc)_3_, (GlcNAc)_4_, and (GlcNAc)_5_ were found (Fig. 4f). Finally, (GlcNAc)_2_–(GlcNAc)_6_ were both hydrolyzed to GlcNAc as the final product with the increase of hydrolysis time. Similar results were reported for GH20 NAGase (*Vh*Nag2) from *V. harveyi* [59] and *Le*Hex20A from *L. edodes* [58]. However, the hydrolysis rates of *Vh*Nag2 and *Le*Hex20A toward *N*-Acetyl COSs were in the order: (GlcNAc)_4_ > (GlcNAc)_3_ > (GlcNAc)_5_ > (GlcNAc)_6_ > (GlcNAc)_2_. Based on these results, we conclude that *Cm*NAGase is a typical exo-acting NAGase.

In addition, minor (GlcNAc)_3_, (GlcNAc)_4_, (GlcNAc)_5_, (GlcNAc)_6_, and new peak generated after the peak of (GlcNAc)_6_ were also produced from (GlcNAc)_2_, (GlcNAc)_3_, (GlcNAc)_4_, (GlcNAc)_5_, and (GlcNAc)_6_ in short reaction times, respectively (Fig. 4b–f). The mass spectrum analysis of the new peak is at *m*/*z* values of 1440.5756 and 1441.5743, which respectively correspond to (GlcNAc)_7_ and (GlcNAc)_7_ with a hydrogen adduct (Additional file 1: Fig. S4). These results showed that *Cm*NAGase can produce higher *N*-Acetyl COSs (GlcNAc)_3_–(GlcNAc)_7_ from (GlcNAc)_2_–(GlcNAc)_6_, respectively. To date, most scholars all believe that production of (GlcNAc)_*n*+1_ from (GlcNAc)_*n* (*n*=2–7)_ is a transglycosylation reaction [28, 29, 34]. However, it may also be a reverse hydrolysis reaction. For example, (GlcNAc)_2_ and (GlcNAc)_*n*_ may form (GlcNAc)_*n*+1_ by transglycosylation. Meanwhile, GlcNAc and (GlcNAc)_*n*_ could also form (GlcNAc)_*n*+1_ through reverse hydrolysis. It is very hard to tell the two reactions apart in this setup. Thus, we call this reaction transglycosylation for the time being.

To date, some chitinases have been used to synthesize higher *N*-Acetyl COSs from shorter *N*-Acetyl COSs substrates via transglycosylation activity. For example, a chitinase from *C. shinanonensis* generated (GlcNAc)_5_ and (GlcNAc)_6_ when incubated with (GlcNAc)_4_ [62]. A chitinase from *T. reesei* KDR-11 was shown to convert (GlcNAc)_4_ into (GlcNAc)_6_ [63]. An endochitinase of *Flavobacterium johnsoniae* synthesized (GlcNAc)_6_–(GlcNAc)_8_ and (GlcNAc)_7_–(GlcNAc)_9_ from (GlcNAc)_5_ and (GlcNAc)_6_, respectively [64]. A chitinase from *Microbulbifer thermotolerans* DAU221 produced (GlcNAc)_4_ from (GlcNAc)_3_ [65].

For GH20 NAGases, production of higher *N*-Acetyl COSs from shorter *N*-Acetyl COSs was often catalyzed by an auto-condensation reaction (a special case of transglycosylation, which involves only one substrate (acts both donor and acceptor) [24]. For example, a GH20 NAGase from *A. oryzae* was used to catalyze the formation of (GlcNAc)_3_ and (GlcNAc)_4_ from (GlcNAc)_2_ [29]. Singh et al. [28] reported that the GH20 NAGase from *A. oryzae* produces (GlcNAc)_4_–(GlcNAc)_6_ and (GlcNAc)_5_–(GlcNAc)_6_ from (GlcNAc)_3_ and (GlcNAc)_4_, respectively. GH20 NAGases *Sm*Hex from *S. marcescens* YS-1 [35] and *No*Hex from *N. orientalis* IFO12806 [66] were shown to convert (GlcNAc)_2_ to (GlcNAc)_3_. GH20 NAGases *Ao*Hex from *A. oryzae* CCF1066 produce the mixture of (GlcNAc)_2_–(GlcNAc)_8_ from the mixture of GlcNAc–(GlcNAc)_7_ [34]. In comparison with these reports, *Cm*NAGase is a novel bacteria-derived NAGase, which possesses transglycosylation activity toward (GlcNAc)_2_–(GlcNAc)_6_.

In addition, the products ((GlcNAc)_3_–(GlcNAc)_7_) from transglycosylation disappeared soon with the increase of reaction time (Fig. 4b–f). This phenomenon may be because the NAGase activity of *Cm*NAGase outweighs its transglycosylation activity, which leads to the transient existence of (GlcNAc)_3_–(GlcNAc)_7_.

### Reverse hydrolysis activity of recombinant *Cm*NAGase toward GlcNAc

**Fig. 5:**
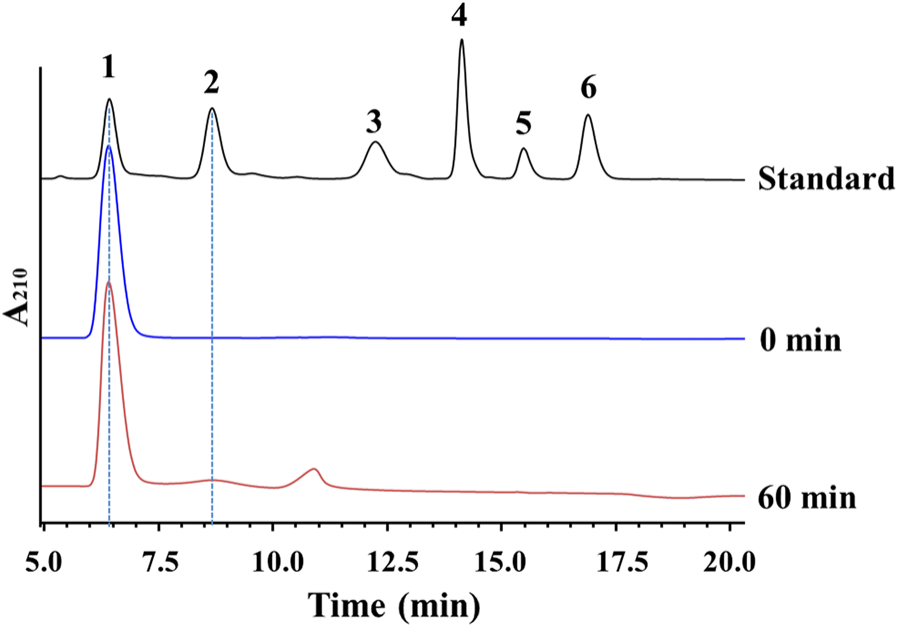
HPLC analysis of the products from GlcNAc by reverse hydrolysis activity of *Cm*NAGase. GlcNAc of 10 g/L was incubated with *Cm*NAGase (3 μg) in 1 mL volume at pH 5.4 and 35 °C for different time intervals. 1–6: Standards of GlcNAc, (GlcNAc)_2_, (GlcNAc)_3_, (GlcNAc)_4_, (GlcNAc)_5_, and (GlcNAc)_6_

In view of the transglycosylation activity toward (GlcNAc)_2_–(GlcNAc)_6_, the reverse hydrolysis activity of *Cm*NAGase was also investigated using GlcNAc as the substrate. As shown in Fig. 5, two new peaks at 8.2 min (peak 1) and 10.9 min (peak 2) were detected by HPLC. Of these, peak 1 was (GlcNAc)_2_ compared with the standard of GlcNAc–(GlcNAc)_6_. However, the retention time of peak 2 was between that of (GlcNAc)_2_ (8.2 min) and (GlcNAc)_3_ (12.3 min). To further identify peak 2, mass spectrum analysis was conducted. The *m*/*z* value of peak 2 was at 447.1586, which corresponds to (GlcNAc)_2_ (425.1766 Da) with a sodium adduct (22.9898 Da) (Additional file 1: Fig. S5). The result showed that peak 2 was also GlcNAc dimer.

The reverse hydrolysis reaction of GH20 NAGases toward GlcNAc can form many connection configurations of the GlcNAc dimer, for example by β(1 → 3), β(1 → 4), and β(1 → 6) glycosidic bonds, which is mainly determined by the regioselectivity of glycosidase [30]. To date, studies on the synthesis of GlcNAc dimer from GlcNAc were mainly reported by Rauvolfová et al., which produce β-GlcNAc-(1 → 3)-GlcNAc, β-GlcNAc-(1 → 4)-GlcNAc, and β-GlcNAc-(1 → 6)-GlcNAc using the library of fungal GH20 NAGases [30]. For example, the GH20 NAGases from *Acremonium persicinum* CCF 1850, *A. oryzae* CCF 1066, *Aspergillus flavipes* CCF 2026, *A. flavus* CCF 1129, *P. oxalicum* CCF 2315, and *A. terreus* CCF 2539, which only produced β-GlcNAc-(1 → 6)-GlcNAc from GlcNAc [30, 31]. The GH20 NAGases from *P. funiculosum* CCF 1994, *P. funiculosum* CCF 2325, *P. chrysogenum* CCF 1269, and *Trichoderma harzianum* CCF 2687 synthesized β-GlcNAc-(1 → 3)-GlcNAc, β-GlcNAc-(1 → 4)-GlcNAc, and β-GlcNAc-(1 → 6)-GlcNAc from GlcNAc [30]. The GH20 NAGases from *A. fumigatus* CCF 1059, *P. pittii* CCF 2277, and *A. sojae* CCF 3060 synthesized β-GlcNAc-(1 → 4)-GlcNAc and β-GlcNAc-(1 → 6)-GlcNAc from GlcNAc. Among these fungal NAGases, β-GlcNAc-(1 → 6)-GlcNAc is always the main product. Thus, the peak 1 in Fig. 5 was β-GlcNAc-(1 → 4)-GlcNAc ((GlcNAc)_2_), and main peak 2 should be β-GlcNAc-(1 → 6)-GlcNAc. These results suggested that *Cm*NAGase has reverse hydrolysis activity toward GlcNAc.

## Conclusions

This study reports the isolation, cloning, and recombinant expression of the gene encoding *Cm*NAGase from *C. meiyuanensis* SYBC-H1. *Cm*NAGase contains a GH20 family catalytic module and exhibits low similarity with reported GH20 NAGases. Analysis of the hydrolysis products from *N*-Acetyl COSs and colloidal chitin revealed that *Cm*NAGase exhibited exo-acting activity. Interestingly, *Cm*NAGase possesses transglycosylation activity toward (GlcNAc)_2_–(GlcNAc)_6_, which respectively leads to synthesis of (GlcNAc)_3_–(GlcNAc)_7_. In addition, *Cm*NAGase also have reverse hydrolysis activity toward GlcNAc, which can produce its dimers with different linked. This is first report of a bacterial NAGase, which can produce GlcNAc dimers from GlcNAc via reverse hydrolysis activity.

## Methods

### Chemicals

Chitin, 4-Methylumbelliferyl *N*-Acetyl glucosaminide (4-MU-GlcNAc), *p*-Nitrophenyl *N*-Acetyl glucosaminide (*p*NP-GlcNAc), *p*NP-glucose, and *p*NP-acetyl galactosaminide were purchased from Aladdin reagent Co., Ltd (Shanghai, China). The standards of *N*-Acetyl chitooligosaccharides (*N*-Acetyl COSs) (purity: ≥ 95%) with degree of polymerization between 2 and 6 were acquired from Qingdao Bozhi Biotechnology Co., Ltd (Qingdao, China). Peptone and yeast extract were purchased from the Oxoid Co., Ltd. (Beijing, China). All molecular reagents were purchased from TaKaRa (Dalian, China). Colloidal chitin was prepared as described by Gao et al. [13]. Other chemicals and solvents used in this study were purchased from local suppliers and were of analytical grade.

### Strains, culture conditions, and plasmids

The *C. meiyuanensis* strain SYBC-H1 (ATCC BAA-2140) used in this study was isolated previously [37]. SYBC-H1 was cultivated according to our previous study [67]. The supernatant was collected as crude enzyme by centrifugation at 6000×*g* at 4 °C and used for NAGase purification.

The strains, plasmids, and primers used in this study are listed in Additional file 1: Table S1. *E. coli* strains were routinely cultivated aerobically at 37 °C in LB medium (10 g/L tryptone, 5 g/L yeast extract, and 5 g/L NaCl). *E. coli* transformants were grown in LB medium or on agar plates containing 50 μg/mL kanamycin.

### Purification of wild-type NAGase from *C. meiyuanensis* SYBC-H1

NAGase was purified by saturation with ammonium sulfate, followed by anion exchange chromatography, and all purification procedures were carried out at 4 °C. The supernatant of the culture was used as a crude enzyme preparation and then fractionated at 40% to 60% saturation with ammonium sulfate. The precipitate was centrifuged at 12,000*g* for 30 min and dissolved in a suitable volume of 50 mM PBS (pH 7.0). The enzyme obtained in the previous step was further purified using a fast protein liquid chromatography (FPLC) system (AKTA Pure 150; GE healthcare Co., Fairfield, USA) with a DEAE Sepharose™ anion exchange column. The column was equilibrated with 50 mM Tris–HCl at pH 8.0, then protein was separated by gradient elution with NaCl solutions from 0.05 to 0.5 M. The purified NAGase was concentrated and collected using an ultrafiltration tube (10 kDa, Millipore, USA) at 4 °C. Then the purified enzyme was analyzed by sodium dodecyl sulfate–polyacrylamide gel electrophoresis (SDS-PAGE) with a 3% stacking gel and a 10% separating gel, according to the method described by Laemmli [68].

After electrophoresis, the gel was sliced vertically into two parts for staining and zymogram analysis. One part was stained using 0.1% Coomassie brilliant blue R-250 and then decolorized with a mixture of 10% acetic acid, 30% methanol, and 60% water. The other part was incubated with 2.5% (vol/vol) Triton X-100 for 30 min twice to refold, then was sprayed with 50 mM PBS (pH 7.0) containing 1 mM 4-MU-GlcNAc and incubated at 37 °C for 30 min; the NAGase strip became visible as fluorescence at 340 nm. Two parts were compared to determine the position of the *Cm*NAGase, and the corresponding band was sliced for peptide mass fingerprinting (PMF) analysis.

### Peptide mass fingerprinting of the enzyme

The gel sliced above was analyzed using the electrospray ionization quadrupole time-of-flight mass spectrometer (ESI-Q-TOF MS/MS) technique (PROTTECH, Inc., Suzhou, China). These masses were then compared to theoretical mass values in the Mascot website databases (http://www.matrixscience.com) to reveal the amino acid sequences of the peptide fragments. The peptide sequence was then aligned with the genome of *C. meiyuanensis* SYBC-H1 (GenBank Accession number, CP041335) to find the NAGase and its coding gene.

### Cloning of the *Cm*NAGase gene and sequence analysis

The genomic DNA of *C. meiyuanensis* SYBC-H1 was used as the template for polymerase chain reaction (PCR) amplification. According to the results of PMF and the complete genome of *C. meiyuanensis* SYBC-H1, the coding region of the *Cm*NAGase gene was amplified by PCR with the primer pair *CmNAGase*-F-5′-GAATTCCATATGATGAGCCGTCCCGCCGGATC-3′ and *CmNAGase*-R-5′-TCCGCTCGAGTCAGGCGCCCACCTGCACCG-3′. The PCR conditions were as follows: 5 min at 94 °C, followed by 30 cycles of 95 °C for 30 s, 60 °C for 30 s, and 72 °C for 10 min. The amplified PCR product was purified by gel electrophoresis, digested with restriction enzymes NdeI and XhoI, and then ligated into pMD19-T Simple vector and sequenced by Invitrogen Corporation (Shanghai, China). The positive recombinant plasmids were digested with NdeI and XhoI, and the gene was inserted into the pET-28a(+) vector expression plasmid with a C-terminal His6-tag to generate the pET-28a(+)-*CmNAGase.*

Nucleotide and amino acid sequences were analyzed using Snap Gene™ 1.1.3 software and the ExPASy Protparam tool (http://web.expasy.org/protparam/) [69]. The conserved domains and the GH family classification were identified via the SMART website (http://smart.embl-heidelberg.de/) [70]. The DNA and protein sequence alignments were performed via the NCBI server with the programs BLASTN and BLASTP (http://blast.ncbi.nlm.nih.gov/Blast.cgi) [71], respectively. Phylogenetic trees were inferred using neighbor-joining algorithm in MEGA 7.0 software and assessed using 1000 bootstrap replications. The presence of a signal peptide and enzyme location were analyzed using the SignalP 5.0 server (http://www.cbs.dtu.dk/services/SignalP/) [72] and Gneg-mPLoc server v.2.0 (http://www.csbio.sjtu.edu.cn/bioinf/Gneg-multi/) [73]. Protein homologous sequences’ alignment was carried out using ClustalX 2.1 software and ESPript 3.0 (http://espript.ibcp.fr/ESPript/cgi-bin/ESPript.cgi) [74]. Three-dimensional (3D) structure of *Cm*NAGase was predicted with RaptorX (http://raptorx.uchicago.edu/StructPredV2/predict/) [75].

### Expression of the *Cm*NAGase gene in *E. coli* BL21(DE3) and purification of the recombinant enzyme

The recombinant plasmid pET-28a(+)-*CmNAGase* above was transformed into competent *E. coli* BL21(DE3) for protein expression. The *E. coli* BL21(DE3) harboring the pET-28a(+)-*CmNAGase* plasmid were cultured in LB medium (containing 50 μg/mL kanamycin) at 37 °C in a shaker with a rotation speed of 200 rpm. When the optical density (OD_600_) of the culture medium reached 0.6–0.8, isopropyl β-d-thiogalactoside (IPTG) was added at a final concentration of 1 mM for protein induction, and the culture was further grown at 25 °C for 12 h.

The cells were harvested by centrifugation at 6000*g* and 4 °C for 10 min, after which the cells were re-suspended with His6-tag binding buffer (20 mM Tris–HCl, 500 mM NaCl, 50 mM imidazole [pH 7.0]) and lysed by JY92-IIN ultrasonication (Ningbo Xinzhi Biotechnology, Ltd., Ningbo, China). Cell debris was removed by centrifugation at 6000*g* for 10 min at 4 °C and the supernatant was retained as crude enzyme. The recombinant *Cm*NAGase were purified using an FPLC system (AKTA Pure 150; GE healthcare Co., Fairfield, USA)) with a Ni-nitrilotriacetic acid affinity chromatography (Ni-NTA) column (His Trap™ FF 5 mL). The target protein was eluted with elution buffer (20 mM Tris–HCl, 500 mM NaCl, 250 mM imidazole [pH 7.0]). The eluted fractions were passed through an ultrafiltration tube of 10 kDa (Millipore, USA) to remove the imidazole with sodium phosphate buffer (pH7.0) and concentrate the enzyme solution.

### Determination of protein concentration and molecular weight

Concentration of protein was quantified using the Bradford method [76]. Bovine serum albumin (BSA) was used to construct a standard calibration curve.

Reductive SDS-PAGE with a 3% stacking gel and 10% separating gel was performed to determine the molecular weight of purified recombinant protein according to purification part of wild-type NAGase above. A premixed protein marker (Takara Biotechnology Co., Ltd., Nanjing, China) containing 180-, 140-, 100-, 75-, 60-, 45-, 35-, 25-, 15-, and 10-kDa bands was used as the molecular mass standard.

### Determination of enzymatic activity

The NAGase activity for *Cm*NAGase used *p*NP-GlcNAc as the substrate [77]. A total of 20 μL of the enzyme solution (0.1 g/L) was added to 0.98 mL *p*NP-GlcNAc (0. 25 mM) in 50 mM sodium citrate buffer (pH 5.4) and incubated at 40 °C for 10 min. The reaction was terminated by adding 2 mL NaOH (0.5 M). The absorbance was measured at 405 nm to determine the amount of *p*NP produced using a standard curve. One unit of NAGase activity was defined as the amount of enzyme required to release 1 μmol *p*NP from the substrate per minute at 40 °C.

### Characterization of recombinant *Cm*NAGase

With 1 mM *p*NP-GlcNAc as the substrate, the optimum pH for activity of *Cm*NAGase was determined using different buffers: 50 mM citrate buffer (pH 3.0–6.0), 50 mM phosphate buffer (pH 6.0–7.5), and Tris–HCl buffer (pH 7.0–9.0) at 40 °C for 30 min. To measure the pH stability, enzyme was incubated at 35 °C for 96 h in the different buffers and the residual activities were determined against 1 mM *p*NP-GlcNAc.

The optimum temperature of *Cm*NAGase activity was measured, and the reaction solutions were incubated at temperatures that ranged from 25 to 55 °C for 30 min. Enzyme thermostability was determined by measuring the residual activities after pre-incubation of the purified enzyme in 50 mM sodium citrate buffer (pH 5.4) at 20–40 °C without substrate for 12 h. The residual activities were performed at pH 5.4 and 40 °C according to enzymatic activity assay above.

The effects of metal ions on the activity were also determined. Purified recombinant *Cm*NAGase was treated with 10 mM EDTA for 4 h at 4 °C and then dialyzed against 50 mM Tris–HCl buffer (pH 7.0) to remove the EDTA. For reactivation, the metal-free enzyme was incubated with various metal salts containing Cr^3+^ (CrCl_3_), Fe^3+^ (FeCl_3_), Fe^2+^ (FeCl_2_), Ca^2+^ (CaCl_2_), Cu^2+^ (CuCl_2_), Mg^2+^ (MgCl_2_), Zn^2+^ (ZnCl_2_), Mn^2+^ (MnCl_2_), Ni^2+^ (NiCl_2_), Co^2+^ (CoCl_2_), K^+^ (KCl), or Na^+^ (NaCl) at final concentrations of 10 mM for 30 min, and the residual activities were then measured with *p*NP-GlcNAc at 40 °C and pH 7.0 (Tris–HCl buffer) for 10 min. The activity without addition of metal ions was used as the control (100%).

The substrate specificity of *Cm*NAGase was determined by measuring the activity of enzyme toward colloidal chitin, *N*-acetyl COSs ((GlcNAc)_2_–(GlcNAc)_6_), cellobiose, 4-MU-GlcNAc, and *p*NP-glycosides as substrates) at a concentration of 10 mg/mL. Reaction mixture (1 mL) containing enzyme (3 μg) and various substrates (10 g/L) was incubated at 40 °C and pH 5.4 for 10 min. The amount of reducing sugars from colloidal chitin and (GlcNAc)_2_–(GlcNAc)_6_ was quantified with HPLC. The amount of *p*NP released in the reaction mixture was determined by measuring the absorbance at 405 nm. Hydrolytic activity of the enzyme against *N*-acetyl COSs was assayed by measuring the amount of GlcNAc released during the enzymatic reaction. After incubation, the enzymatic reaction was stopped by heating the mixture in a boiling water bath for 5 min. One unit of enzyme activity was defined as the amount of enzyme required to liberate 1 μmol of *p*NP or GlcNAc per minute under the assay conditions.

Kinetics experiments were performed using *p*NP-GlcNAc as the substrate. The initial velocities were determined by incubating 17 ng purified *Cm*NAGase with *p*NP-GlcNAc concentrations ranging from 50 to 1000 μM at 40 °C in a 1 mL reaction system (50 mM sodium citrate buffer, pH 5.4) for 5 min. The values of *V*_max_, *K*_m_, and *K*_cat_ were estimated by linear regression from double-reciprocal plots according to the method of Lineweaver [78].

### Hydrolysis reaction of the recombinant *Cm*NAGase toward colloidal chitin

A 1 mL reaction system (50 mM sodium citrate buffer, pH 5.4) containing colloidal chitin (10 g/L) and purified *Cm*NAGase (3 μg) was conducted at 35 °C for various time intervals. Boiling (5 min) was used to stop the reaction.

### Hydrolysis and transglycosylation reactions of the recombinant *Cm*NAGase toward *N*-acetyl COSs

A 100 μL volume (50 mM sodium citrate buffer, pH 5.4) with 10 g/L *N*-Acetyl COSs ((GlcNAc)_2_–(GlcNAc)_6_) and 0.3 μg purified *Cm*NAGase was conducted at 35 °C for various time intervals. The enzyme reactions were stopped by boiling at 100 °C for 5 min.

### Reverse hydrolysis reaction of the recombinant *Cm*NAGase toward GlcNAc

The determination of reverse hydrolysis activity was performed with 10 g/L GlcNAc and 0.3 μg purified *Cm*NAGase in a 100 μL volume at pH 5.4 and 35 °C for 1 h. The reactions were stopped by boiling at 100 °C for 5 min.

### Analysis of products

The resulting reaction products were analyzed with an Agilent 1260 series HPLC system according to our previous report [77]. The molecular mass of the product was analyzed using electrospray ionization mass spectrometry (ESI-MS, API 2000) with the ESI positive mode. The quadrupole scan mode was used under a capillary voltage of 2.8 kV, cone voltage of 30 V, desolvation gas temperature of 350 °C and source temperature of 120 °C.

### Nucleotide sequence accession number

The sequence for the gene encoding *Cm*NAGase cloned from strain SYBC‑H1 was deposited in GenBank under Accession number no. MN548778.

## Supplementary information

**Additional file 1: Table S1.** Strains, plasmids, and primers used in this study. **Table S2.** Purification of recombinant *Cm*NAGase. **Table S3.** Half-lives of recombinant *Cm*NAGase. **Fig. S1.** Multiple alignments of the catalytic domain in *Cm*NAGase with other GH20 NAGases. **Fig. S2.** The domain and structure prediction of *Cm*NAGase. a) The conserved domain of *Cm*NAGase. b) The prediction of the 3D structure of *Cm*NAGase. c) The active site of *Cm*NAGase. **Fig. S3.** SDS-PAGE analysis of recombinant *Cm*NAGase. **Fig. S4.** Mass spectrum of new peak (~ 16.0 min) after (GlcNAc)_6_ in HPLC spectra. **Fig. S5.** Mass spectrum of peak 2 (~ 10.9 min) in HPLC spectra.

## Data Availability

The datasets used and/or analyzed during the current study are available from the corresponding author on reasonable request.

## Abbreviations

GlcNAc: *N*-Acetyl glucosamine
NAGase: β-*N*-acetyl glucosaminidase
*N*-Acetyl COSs: *N*-Acetyl chitooligosaccharides
GH: Glycoside hydrolase
4-MU-GlcNAc: 4-Methylumbelliferyl *N*-Acetyl glucosaminide
*p*NP-GlcNAc: *p*-Nitrophenyl *N*-Acetyl glucosaminide
PMF: Peptide mass fingerprinting
SDS-PAGE: Sodium dodecyl sulfate–polyacrylamide gel electrophoresis
PCR: Polymerase chain reaction
FPLC: Fast protein liquid chromatography
MOWSE: Molecular weight search scores
CAZy: Carbohydrate-Active enZYmes
