# Supplementary material for: A novel bacterial β-N-acetyl glucosaminidase from Chitinolyticbacter meiyuanensis possessing transglycosylation and reverse hydrolysis activities
Source: Biotechnol Biofuels. 2020 Jun 29;13:115. doi: 10.1186/s13068-020-01754-4 (PMC7324980; doi:10.1186/s13068-020-01754-4)
Supplement: Supplementary file 1 — Additional file 1: Table S1. Strains, plasmids, and primers used in this study. Table S2. Purification of recombinant CmNAGase. Table S3. Half-lives of recombinant CmNAGase. Fig. S1. Multiple alignments of the catalytic domain in CmNAGase with other GH20 NAGases. Fig. S2. The domain and structure prediction of CmNAGase. a) The conserved domain of CmNAGase. b) The prediction of the 3D structure of CmNAGase. c) The active site of CmNAGase. Fig. S3. SDS-PAGE analysis of recombinant CmNAGase. Fig. S4. Mass spectrum of new peak (~ 16.0 min) after (GlcNAc)6 in HPLC spectra. Fig. S5. Mass spectrum of peak 2 (~ 10.9 min) in HPLC spectra. [file 13068_2020_1754_MOESM1_ESM.docx]

**A novel bacterial β-*N*-acetyl glucosaminidase from *Chitinolyticbacter meiyuanensis* possessing transglycosylation and reverse hydrolysis activities**

Alei Zhang, Xiaofang Mo, Ning Zhou, Yingying Wang, Guoguang Wei, Jie Chen, Kequan Chen*, Pingkai Ouyang

*State Key Laboratory of Materials-Oriented Chemical Engineering, College of Biotechnology and Pharmaceutical Engineering, Nanjing Tech University, Nanjing, 211800, P.R. China*

*Corresponding author: Tel.: +86-138-1418-0652

E-mail address: kqchen@njtech.edu.cn

**Table S1** Strains, plasmids, and primers used in this study

| Strain, plasmid,  or primer | Description*^a^* | Source |
| --- | --- | --- |
| Strains  *E.coli* DH5α  *E.coli* BL21(DE3)  Strain SYBC-H1 | *ϕ*80d*lac*ZΔM15 Δ(*lacZY-argF*)U169 *deoR recA*1 *endA1 hsdR17*(r_k_^-^ m_k_^+^) *supE44 thi*-1 *gyrA96 relA1*  F^-^ *ompT hsdS_B_(r_B_^-^m_B_^-^) gal dcm* (DE3)  Wild type, isolated from soil | Invitrogen  Novagen  This study |
| Plasmids  pMD19-T simple  pET28a(+)  pMD19-*CmNAGase*  pET28a(+)-*CmNAGase* | *E.coli* cloning vector; Amp^r^  *E.coli* expression vector,T7 RNA polymerase gene promoter and terminator; Kan^r^  pMD19-T simple derivate, containing the *Cm*NAGase gene from SYBC-H1 strain  7.1-kb pET28a(+) derivate carrying the *Cm*NAGase gene | TaKaRa  Novagen  This study  This study |
| Primers  F1  R1 | GAATTCCATATGATGAGCCGTCCCGCCGGATC  TCCGCTCGAGTCAGGCGCCCACCTGCACCG |  |

*^a^*Underlined sequences within the primers are the *Nde*I and *Xho*I restriction sites. Amp^r^, ampicillin resistance; Kan^r^, kanamycin resistance.

**Table S2** Purification of recombinant *Cm*NAGase.

| Purification method | Total activity  (U) | Total protein  (mg) | Specific activity (U/mg) | Purification (fold) | Recovery yield (%) |
| --- | --- | --- | --- | --- | --- |
| Crude enzyme | 60,604.2 | 19.2 | 3,156.5 | 0 | 100 |
| Ni–NTA resin | 48,786.4 | 10.4 | 4,878.6 | 1.5 | 80.5 |

**Table S3** Half-lives of recombinant *Cm*NAGase.

| Temperature (ºC) | Half-life (h) |
| --- | --- |
| \| 30 \| \| --- \| | 13.0 ± 0.82 |
| \| 35 \| \| --- \| | 9.5 ± 0.35 |
| 40 | 6.3 ± 0.39 |
| \| 45 \| \| --- \| | 0.6 ± 0.01 |

**Fig. S1** Multiple alignments of the catalytic domain in *Cm*NAGase with other GH20 NAGases from different sources. Similar sequences are marked by boxes and identical sequences are highlighted in red and yellow. The H/N-X-A/C/G/M-D-E-A/I/L/V motifs are marked with a black box. The conserved substrate binding and catalytic residues are marked with a red hashtag. Secondary structural elements (i.e., alpha helix [α], beta sheet [β], random coil [ƞ], and beta turn [T]) are marked on the *Cm*NAGase sequence. WP_018749679: NAGase from *Chitiniphilus shinanonensis* (WP_018749679); BAA92145: NAGase from *Aeromonas* sp. 10S-24 (accession no. BAA92145); 1C7T_A : NAGase from *Serratia marcescens* (PDB no. 1C7T_A); P13670: NAGase from *Vibrio harveyi* (accession no. P13670); NP_719056: NAGase from *Shewanella oneidensis* MR-1 (accession no. NP 719056); BAB17855: NAGase from *Alteromonas* sp. O-7 (accession no. BAB17855); YP_444514: NAGase from *Salinibacter ruber* DSM 13855 (accession no. YP_444514); ZP_01460692 NAGase from *Stigmatella aurantiaca* DW4/3-1.


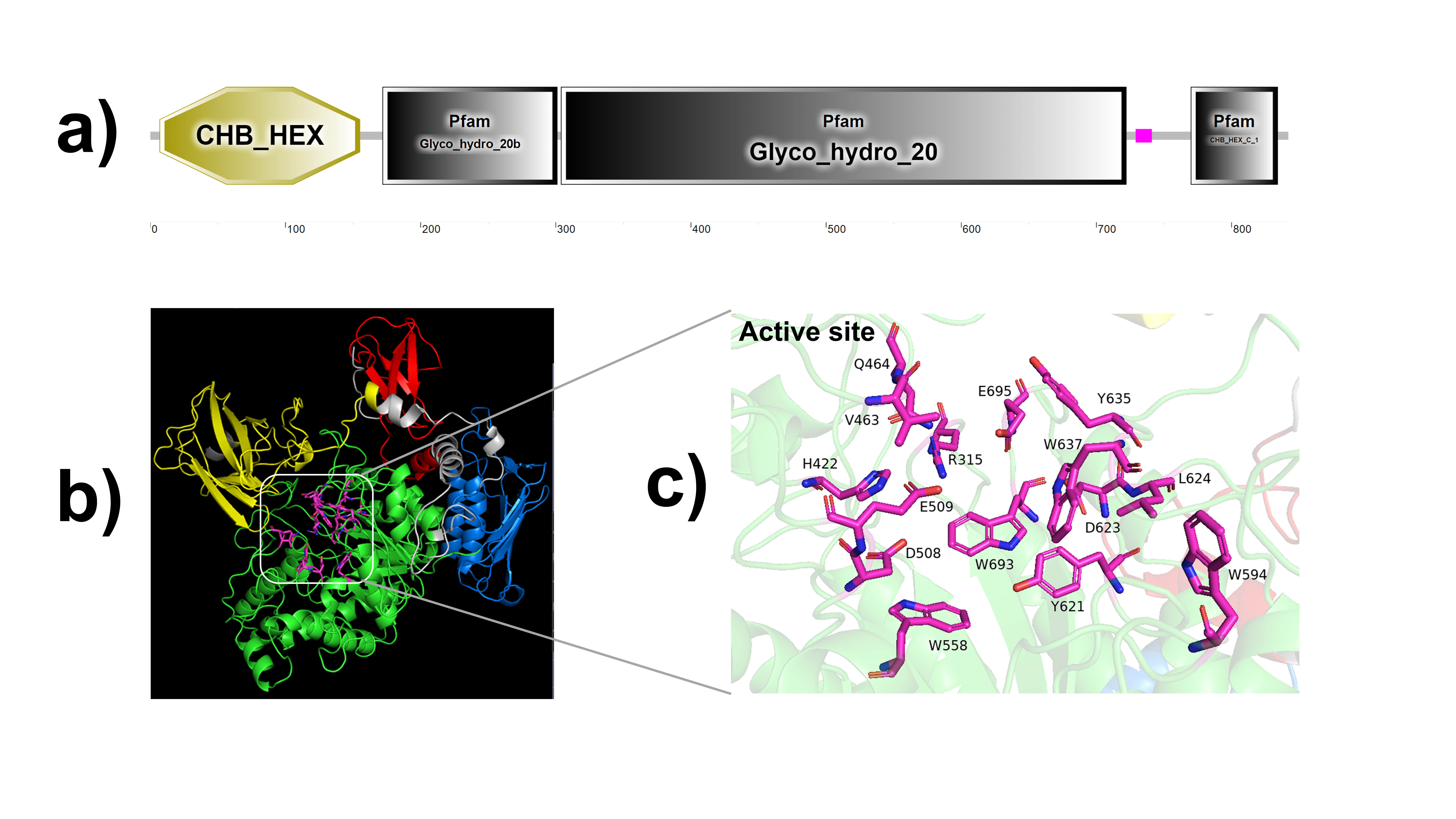


**Fig. S2** The domain and structure prediction of *Cm*NAGase. a) The conserved domain of *Cm*NAGase. b) The prediction of the 3D structure of *Cm*NAGase. Yellow color, CHB_HEX domain; Blue color, Glyco_hydro_20b domain; Green color, Glyco_hydro_20 domain; Red color, CHB_HEX C_1 domain; Gray color, unknow region. c) The active sites of *Cm*NAGase. D508 and E509 were the active residues.

**
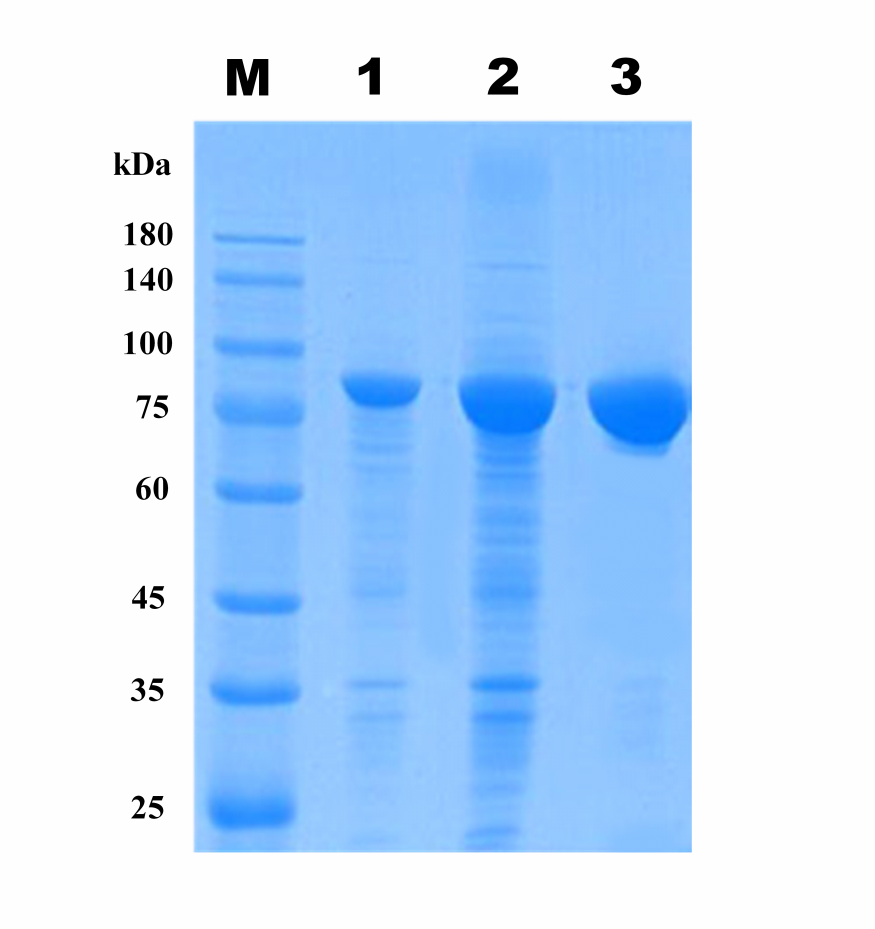
**

**Fig. S3** SDS-PAGE analysis of recombinant *Cm*NAGase. Lane M, protein molecular mass maker; lane 1 and lane 2, 20 μg and 60 μg of soluble protein extracted from *E. coli* cells overexpressing His6-*Cm*NAGase; lane 3, 40 μg of *Cm*NAGase purified by His6-tag affinity chromatography.


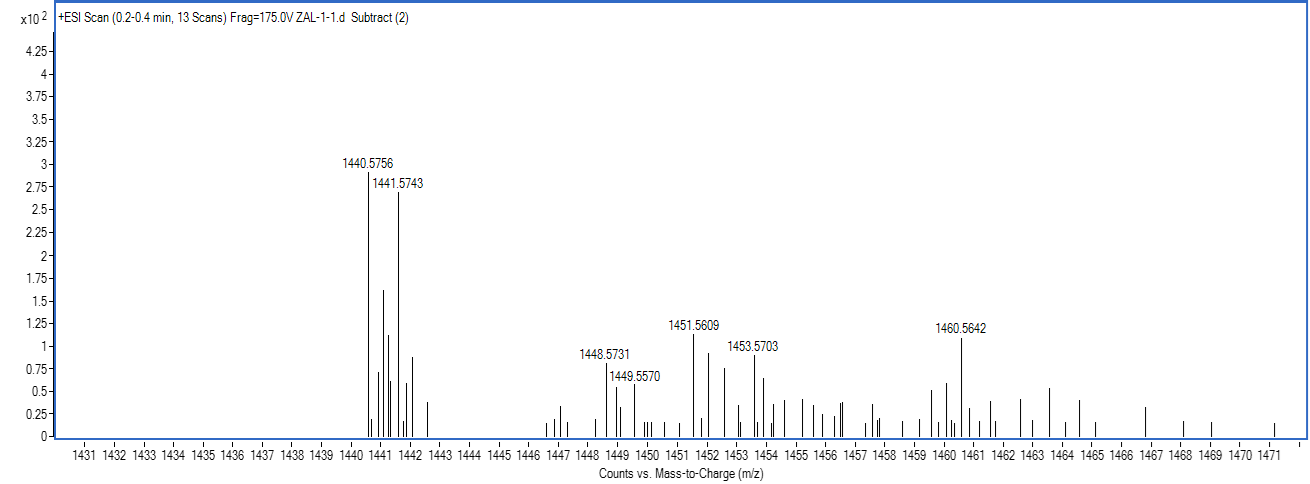


**Fig. S4** Mass spectrum of new peak (~16.0 min) after (GlcNAc)_6_ in HPLC spectra.

**Fig. S5** Mass spectrum of peak 2 (~10.9 min) in HPLC spectra.
